# Membrane Stress Enhances Specific PQS–Lipid Interactions That Drive Bacterial Outer Membrane Vesicle Biogenesis

**DOI:** 10.3390/membranes15080247

**Published:** 2025-08-13

**Authors:** Citrupa Gopal, Hasan Al Tarify, Emad Pirhadi, Eliza G. O’Brien, Anuradha Dagar, Xin Yong, Jeffrey W. Schertzer

**Affiliations:** 1 Department of Biological Sciences, Binghamton University, Binghamton, NY 13902, USA; cgopal1@binghamton.edu (C.G.); eobrie17@binghamton.edu (E.G.O.); 2 Department of Mechanical Engineering, Binghamton University, Binghamton, NY 13902, USA; altar012@umn.edu (H.A.T.); emadpirh@buffalo.edu (E.P.); xinyong@buffalo.edu (X.Y.); 3 Department of Mechanical and Aerospace Engineering, University at Buffalo, Buffalo, NY 14260, USA; 4 Department of Chemistry, Binghamton University, Binghamton, NY 13902, USA; fanuradh@binghamton.edu

**Keywords:** outer membrane vesicles, *Pseudomonas aeruginosa*, *Pseudomonas* quinolone signal, Bilayer-Couple, molecular dynamics simulation, free energy analysis, membrane stress

## Abstract

Gram-negative bacteria use outer membrane vesicles (OMVs) for toxin trafficking, immune interference, horizontal gene transfer, antibiotic protection, and cell–cell communication. Despite their direct contribution to many pathogenesis-related behaviors, our understanding of how OMVs are produced remains surprisingly incomplete. The Bilayer Couple model describes the induction of OMV formation resulting from the preferential accumulation of small molecules in the outer leaflet of the membrane, resulting in leaflet expansion and membrane bending. Previous work has highlighted the importance of the structure of the *Pseudomonas* Quinolone Signal (PQS) in driving OMV formation, but the nature of interactions with membrane lipids remains unclear. Our recent in silico analysis suggested that a new interaction, between the PQS ring nitrogen and Lipid A, is critical for PQS function. Here, we used chemical analogs to interrogate the importance of specific PQS functional groups in its ability to stimulate OMV biogenesis. We demonstrated that OMV induction requires the presence of all PQS functional groups together. Further modeling uncovered that PQS prefers interaction with the outer leaflet of the membrane, consistent with its unique ability to drive OMV biogenesis. This was explained by much greater hydrogen bond formation between PQS and Lipid A. Interestingly, the preference of PQS for the outer leaflet coincided with that leaflet becoming crowded. Thus, the initial insertion of PQS into the outer leaflet would be expected to encourage local accumulation of more PQS to drive the induction of membrane curvature and subsequent OMV formation.

## 1. Introduction

Outer membrane vesicles (OMVs) are small unilamellar structures, typically 50–250 nm in diameter, that bud from the outer membrane (OM) of all Gram-negative species [1]. They play critical roles in virulence and other community interactions across species [2,3,4,5], and have been described as a dedicated secretion system in bacteria [6,7]. The ability to package and deliver virulence factors [8,9,10,11], nucleic acids [12,13,14,15], communication factors [16,17,18], and inflammatory lipids [19,20] to both bacteria and host cells has led to the association of OMVs with direct pathogenicity [21,22,23,24], immune evasion and modulation [25,26,27,28], protection against antibiotics [29,30,31,32], horizontal gene transfer [33,34,35], and biofilm development [36,37,38]. Despite their clinical relevance, the mechanisms of OMV biogenesis remain incompletely understood.

Based on studies performed using a range of organisms, several models of OMV biogenesis have been proposed [2,3,39]. These include the loss of OM anchoring to peptidoglycan [40,41,42], lipid remodeling in the OM [43,44], responses to stress [45,46], and outright cell lysis [47]. Our own work using *P. aeruginosa* as a model has uncovered a mechanism where OMV formation is increased through interactions of a self-produced small molecule with the outer membrane. The Bilayer Couple model [48] describes preferential insertion of the *Pseudomonas* Quinolone Signal (PQS) into the outer leaflet of the outer membrane, leading to asymmetric expansion of the outer vs. the inner leaflet, and resulting in the induction of membrane curvature. In a broader context, previous studies have demonstrated that the asymmetric insertion or adsorption of “particles”, which may be small molecules, ions, or peptides, can alter the local stress distribution across the bilayer and promote membrane remodeling [49]. Notably, in synthetic giant unilamellar vesicles (GUVs), the asymmetric adsorption of calcium ions has been shown to induce tubular protrusions [50,51]. Similarly, the unequal distribution of ganglioside GM1 between the leaflets of a POPE vesicle can trigger membrane invagination [52]. Computational studies further support that asymmetric localization of small molecules leads to significant variations in membrane stress state [53,54]. This imbalance in stress between the two leaflets modifies the membrane’s spontaneous curvature, providing a physical driving force for shape remodeling such as bending, budding, or vesiculation [55,56].

Support for the Bilayer Couple model comes from biophysical measurement of PQS preference for lipopolysaccharide vs. phospholipid [57], the requirement for PQS export in order to stimulate OMV formation [58], and the ability of PQS to stimulate OMV formation in multiple other species [59,60]. Further, limited structure–function analysis has shown that the presence of the 3-hydroxyl and 2-heptyl groups of PQS are necessary for OMV stimulation, providing insight into why the structurally similar PQS precursor HHQ does not induce OMV production [61]. Previous studies revealed that hypovesiculating mutants are difficult to find [62], but that the loss of PQS synthesis produces a strong hypovesiculation phenotype [16]. Thus, characterizing the PQS–lipid interactions that drive OMV biogenesis is critical for us to understand bacterial pathogenesis related to OMVs.

We recently began to investigate PQS–lipid interactions using molecular dynamics simulations in an attempt to gain greater insight [63]. This analysis confirmed the importance of the hydroxyl and alkyl groups of PQS for membrane binding but also revealed an interesting multi-step sequence to the interaction that provided context for the role of each functional group. The hydroxyl group was primarily responsible for the initial docking of the molecule to the membrane. After this, PQS would execute a remarkable structural folding and insertion step to allow for access of the alkyl chain to the acyl chains of the membrane lipids. Most notably, the simulations suggested a critical but previously unknown contribution of the PQS ring nitrogen group, whose interactions with the Lipid A phosphate groups made the insertion of PQS into the membrane possible.

In the current study, we tested the hypothesis that the ring nitrogen group of PQS is required to stimulate increased OMV biogenesis, and we deepened our molecular dynamics analysis to understand how PQS functional groups facilitate preference for the outer leaflet and the role that inter-leaflet tension plays in the mechanism of OMV biogenesis. Using synthetic PQS analogs, we show that loss of any one functional group eliminates the ability to stimulate OMV formation. Potential of mean force (PMF) analysis revealed that PQS is the only molecule that displays a higher affinity for the outer leaflet of the membrane as it becomes compressed. Finally, we show that PQS engages in the largest number of simultaneous hydrogen bonds in the outer vs. inner leaflet, and that this advantage is also amplified when the outer leaflet becomes overcrowded compared to the stress-free state. With this work, we greatly advance our understanding of the molecular interactions that drive OMV induction by PQS. This includes the first recognition that the ring nitrogen group is required for function, and the novel finding that inter-leaflet stress enhances the PQS effect.

## 2. Materials and Methods

### 2.1. Bacterial Strains and Reagents

*Pseudomonas aeruginosa* strain PA14 [64], Δ*pqsA* [65], and Δ*pqsH* [42] were used for the exogenous addition of PQS and its analogs to assess OMV production. Cultures were grown in Lysogenic Miller broth (LB) (Fisher Scientific, Waltham, MA, USA). PQS, 2-heptyl-4-quinolone (HHQ), 2,4-quinolinediol (DHQ), 2-naphthol (2N), and 4-quinolinol (4Q) were all purchased from MilliporeSigma (Burlington, VT, USA). Finally, 3-heptyl-2-naphthol (C7-2N) was synthesized as detailed below.

### 2.2. Synthesis of 3-Heptyl-2-naphthol

The synthesis was carried out in a two-step procedure. The alkylation of 2-methoxy-naphthalene was first carried out to afford 3-heptyl-2-methoxynaphthalene. The product was then deprotected to provide 3-heptyl-2-napthol with an overall yield of 55%. For the alkylation step, n-butyl lithium (2.5 M in hexane (2.2 mmol, 1.1 equiv.) was added to a stirring solution of 2-methoxy-naphthalene (316 mg, 2.0 mmol, 1.0 equiv.) in dry THF (10 mL) at 0 °C under argon. The mixture was stirred at 0 °C for 30 min, followed by reflux for 1 h. A solution of n-heptyl iodide (360 µL, 2.2 mmol, 1.1 equiv.) in dry THF (2 mL) was then added dropwise and refluxing was continued for another 2 h. The cooled mixture was poured onto an ice water bath (20 mL) and saturated NaHCO_3_ (2 × 100 mL), then extracted with ethyl acetate (3 × 5 mL). The reaction mixture was then concentrated under reduced pressure and purified by silica gel column chromatography (EtOAc/hexane = 1:99) to afford 3-heptyl-2-methoxynaphthalene. The residue was further purified by column chromatography using 2% ethyl acetate and hexane as the eluent to afford 471 mg (91%) of colorless oil. For the deprotection step, a mixture of the purified 3-heptyl-2-methoxynaphthalene (256 mg, 1.0 mmol, 1.0 equiv.) and dry pyridine·HCl (138.0 mg, 1.2 mmol) was heated with stirring at 200 °C for 2 h. After being cooled to about 100 °C, the mixture was poured into ice water (2.0 mL) and extracted with benzene (2 × 5 mL). The combined organic layers were washed with water, dried, and evaporated. Distillation of the residue gives a colorless oil. Recrystallization from n-pentane afforded 153 mg (60%) of the colorless desired compound, 3-heptyl-2-naphthol (details and characterization are included in the Supplementary Materials).

### 2.3. Exogenous Addition of PQS and Analogs

Growth flasks were inoculated to a starting OD_600_ of 0.01 from overnight culture and grown at 37 °C with shaking (250 rpm) until OD_600_ reached 0.5. Cultures were then centrifuged at 7000× *g* for 15 min to pellet cells. The supernatant was removed and replaced with 25 mL fresh sterile LB. Cell pellets were resuspended and cultures were added to new flasks before the addition of PQS or its analogs to 20 µM. Cultures were incubated for 4 h at 37 °C with shaking (250 rpm), following which OD_600_ readings were taken and OMVs were harvested.

### 2.4. Isolation of OMVs from Planktonic Cultures

Cultures were centrifuged at 7000× *g* for 15 min to pellet cells, and the supernatant was filtered using a 0.45 uM polyethersulfone syringe filter (Celltreat, Ayer, MA, USA). OMVs were pelleted from 18 mL of filtered supernatant at 209,438× *g* for 90 min at 4 °C in a Thermo Scientific (Waltham, MA, USA), S50-A rotor. OMV pellets were resuspended in 1 mL MV buffer (50 mM Tris, 5 mM sodium chloride and 1 mM magnesium sulfate, pH 7.4).

### 2.5. Quantification of OMVs

OMV production was quantified by Nanoparticle Tracking Analysis (NTA) using the NanoSight NS300 and accompanying software version 3.4.1 (Malvern Panalytical, Malvern, UK). Samples were diluted to achieve 20–100 particles per frame as per manufacturer’s instructions. Three independent 30 s recordings for each sample were taken at room temperature (25 °C) with a camera level of 13. A detection threshold level of 25 was used to limit background noise. The average number of particles/mL per frame was used to calculate the total number of OMVs in each sample, which was normalized to the final OD_600_ value for that sample.

### 2.6. Succinate Dehydrogenase Assay

This assay was a modification of the method detailed by Kasahara and Anraku [66]. SDH activity in OMV samples was tested against a positive control of lysed PA14 cells to ensure that cell lysis/disintegration was not a major cause of extracellular vesicle production during the experiment. PA14 cells (OD_600_ ≈ 0.2) were lysed via sonication (5.25 min at 30% amplitude by a Branson Digital Sonifier (Branson Ultrasonics, Brookfield, CT, USA)). Reactions were carried out in a 96-well plate (Falcon) in a total volume of 200 µL comprising 50 mM Tris-HCl (pH 8.0), 4 mM potassium cyanide (KCN), 0.04 mM 2,6-dichlorophenolindophenol (DCPIP), 0.2 mM phenazine methosulfate (PMS), 40 mM disodium succinate, and 5 µL of the sample. The reaction mixture without sample, DCPIP, or PMS was incubated for 5–10 min at room temperature. Following this, the supernatant (or sonicated control) sample was added to the mixture and allowed to acclimate for 5–10 min. Lastly, DCPIP and PMS were added in this order to initiate the reaction. SDH activity was quantified by measuring the decrease in absorbance at 600 nm over time at 25 °C using a Tecan Infinite M-200 Pro (Tecan, Männedorf, Switzerland). All samples were tested with a minimum of three technical replicates.

### 2.7. PQS Quantification

First, 1 mL from each culture was combined with 1 mL of acidified ethyl acetate (0.1 mL glacial acetic acid per liter of ethyl acetate) and vortexed briefly. The organic phase was removed and evaporated to dryness under nitrogen gas. Dried samples were resuspended in ACS grade methanol (Fisher Scientific). A 10 × 20 cm silica TLC plate (MilliporeSigma, Burlington, VT, USA) was impregnated with ACS reagent-grade potassium phosphate and activated at 100 °C for 1 h. The plate was pre-wetted with the mobile phase (95:5 dichloromethane: methanol) and dried for 15–20 min again at 100 °C before being spotted with standardized concentrations of pure PQS along with the extracted samples. The plate was imaged under long wavelength UV light.

### 2.8. Molecular Dynamic Simulations

Our model *P. aeruginosa* OM was composed of two leaflets with asymmetric compositions. The outer leaflet consisted of hexa-acylated PA14 Lipid A. The phosphate groups in Lipid A were modeled at the protonated state with a net charge of −1 [67]. The inner leaflet was composed of 1-palmitoyl-2-oleoyl-sn-glycerol-3-phosphoethanolamine (POPE) and 1-palmitoyl-2-oleoyl-sn-glycerol-3-phosphoglycerol (POPG) mixed at a ratio of 2.2 to match experimentally measured profiles [68,69]. The bilayer membrane was solvated in water with calcium (Ca^2+^) and sodium (Na^+^) ions as counterions for neutralizing Lipid A and POPG, respectively. The system also includes additional NaCl at 150 mM concentration to mimic physiological conditions. The structural analogs of PQS were constructed using PyMOL by adding and removing functional groups to and from PQS molecules.

We performed all-atom molecular dynamics simulations using the CHARMM36 force field [70] with Ca^2+^ NBFIX [71] and Na^+^ CUFIX [72] to improve the precision of ion–ion and ion–lipid interactions. To ensure consistency with the membrane force field, we used the CHARMM General Force Field [73] to parameterize the force field and partial charge distribution of PQS and the analogs. The initial configurations of the model membrane and PQS analogs were generated using the CHARMM-GUI membrane builder [74]. The simulations in this study were all performed using the GROMACS 2021.3 software package [75]. Following the standard procedure in CHARMM-GUI, we carried out in sequence: an energy minimization, an isothermal–isochoric (NVT) equilibration of 100 ps at the physiological temperature of 310.15 K, and an isothermal–isobaric (NPT) equilibration of 1 ns at 310.15 K and 1 bar with semi-isotropic pressure coupling. The final NPT equilibration was performed for 500–1000 ns using the Parrinello–Rahman barostat [76] and Nosé–Hoover thermostat [77]. The hydrogen-containing bonds were constrained using the LINCS algorithm [78]. The cutoff of short-range van der Waals and electrostatic interactions was set to 1.2 nm, with the neighbor list updated every 20 time steps. The particle-mesh Ewald method [79] was employed to calculate long-range electrostatic interactions. The time step for the leapfrog integrator was set to 2 fs.

### 2.9. Umbrella Sampling of Potential of Mean Force

The umbrella sampling method [80] was adopted to obtain the potential of mean force (PMF) profile for the small molecules interacting with the OM. The molecules were first positioned 4.0 nm above the membrane center of mass (COM) in the z-direction. The membranes were equilibrated for an extra 200 ns after the addition of the small molecules. The reaction coordinate was defined as the COM distance between the membrane and the small molecule in the z-direction. The effective membrane COM was calculated using the weighted sum of all the atoms inside a cylindrical patch of membrane with a radius of 1.5 nm directly beneath the probe molecules [81]. The initial configuration of each sampling window was produced by a steered molecular dynamics (SMD) simulation. The molecule was pulled through the membrane in the negative z-direction at a rate of 0.005 nm·ps^−1^ using a harmonic potential with a force constant of 1000 kJ·mol^−1^·nm^−2^. To cover the entire range of molecule locations in the membrane, we chose 53 umbrella sampling windows with a 0.15 nm spacing between them. Each sampling window was first equilibrated for 10 ns in the NPT ensemble without data collection. The sampling time for each window was then increased by a 10 ns increment to examine the convergence of the PMF profile. Depending on the molecule and the membrane, each window required 70–90 ns of sampling time. The unbiased probability distribution and PMF profile were obtained using the weighted histogram analysis method (WHAM) [82]. The Bayesian bootstrapping method was used to estimate the statistical errors of the PMF profiles [83].

## 3. Results

### 3.1. Exogenous HHQ Is Converted to PQS, Which Stimulates OMV Production in ΔpqsA

**Figure 1:**
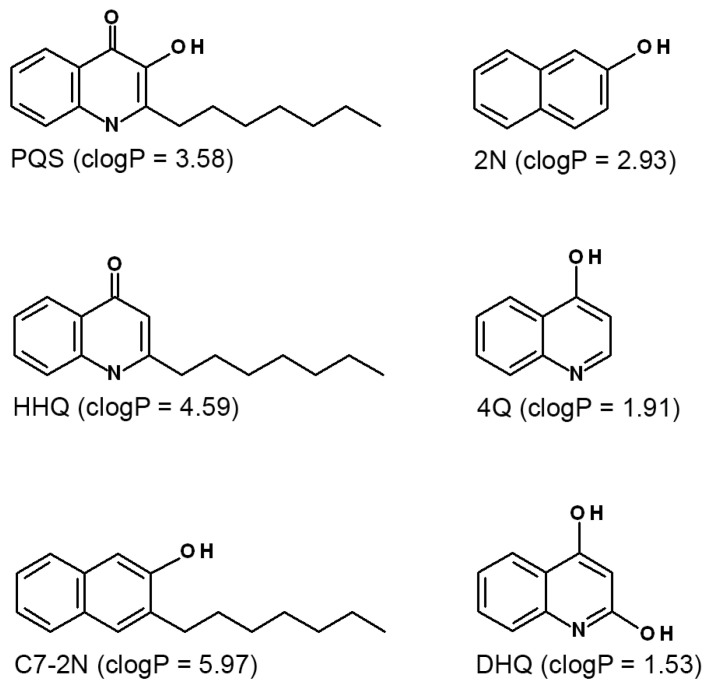
Structures of the small molecules used in this study, along with their clogP values (calculated log octanol–water partition coefficient). clogP values were estimated using ALOGPS 2.1 (http://www.vcclab.org/lab/alogps/start.html (accessed on 10 January 2023)). PQS = 2-heptyl-3-hydroxy-4-quinolone; HHQ = 2-heptyl-4-quinolone; C7-2N = 3-heptyl-2-naphthol; 2N = 2-naphthol; 4Q = 4-quinolinol; DHQ = 2,4-quinolinediol.

**Figure 2:**
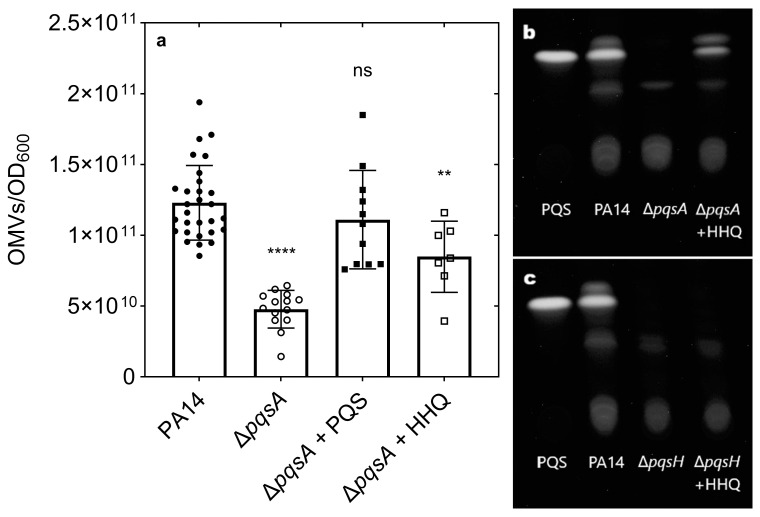
Exogenous addition of PQS and HHQ to Δ*pqsA*. (**a**) Cultures were incubated for 4 h at 37 °C post treatment, followed by OMV harvesting and quantification by NTAnalysis. Error bars represent standard deviation, *n* ≥ 7. Statistical significance was determined by one-way ANOVA followed by Dunnett’s multiple-comparison test against the positive control PA14. ns = not significant, *p* < 0.01 = **, *p* < 0.0001 = ****. Thin-layer chromatography was performed on extracts from (**b**) Δ*pqsA* exposed to HHQ and (**c**) Δ*pqsH* exposed to HHQ. Wild-type PA14 is shown as the positive control, with untreated Δ*pqsA* and Δ*pqsH* strains as the negative controls.

To investigate the contribution of the PQS ring nitrogen to OMV stimulation, we first synthesized 3-heptyl-2-naphthol, an analog of PQS missing the ring nitrogen but retaining both the hydroxyl and heptyl functional groups (Figure 1). We also acquired commercially available analogs displaying various combinations of these three groups (Figure 1). We then set out to expose a PQS-null mutant to the analogs to test their OMV production response. Δ*pqsA* is a biosynthetic mutant strain that lacks anthranilate-CoA ligase activity [84] and is therefore incapable of producing PQS on its own [85]. However, when we exposed this strain to physiologically relevant concentrations of the PQS precursor HHQ, it responded with an intermediate level of OMV production (Figure 2a). Since HHQ is known to lack OMV inducing capability [61], this condition was intended to serve as a negative control for OMV induction. When we analyzed the Δ*pqsA* + HHQ culture by TLC, we identified the production of PQS (Figure 2b). Since the Δ*pqsA* mutant still retains a functional *pqsH* gene (located outside of the *pqsABCDE* operon and coding for the HHQ-hydroxylase enzyme [86]), we reasoned that exogenous HHQ must have been internalized by the cell and converted to PQS by PqsH, which then resulted in stimulation of OMV biogenesis. We repeated the HHQ exposure experiment, this time using the Δ*pqsH* mutant as the recipient strain, and observed no production of PQS (Figure 2c). The exposure of Δ*pqsA* to the remaining analogs resulted in no stimulation of OMV production (Figure S1). To ensure that cell lysis was not a major contributor to OMV production upon the addition of any of the analogs, we tested for the activity of inner membrane protein succinate dehydrogenase (SDH) in all purified OMV preparations and confirmed that no activity was present (Figure S2a).

### 3.2. The Presence of All PQS Functional Groups Is Required for OMV Stimulation

**Figure 3:**
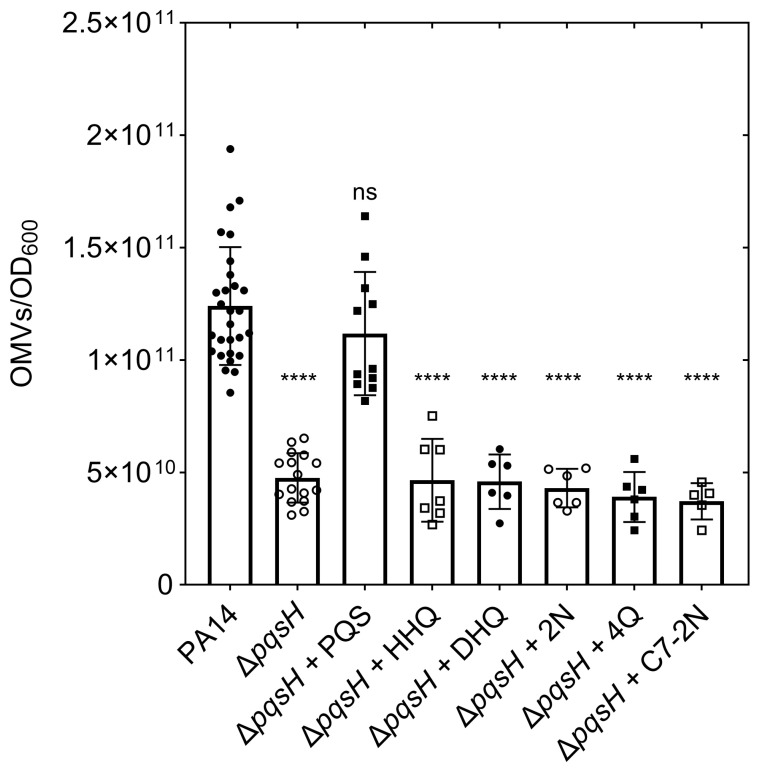
Exogenous addition of PQS analogs to Δ*pqsH*. Cultures were incubated for 4 h at 37 °C post treatment, followed by OMV harvesting and quantification by NTA. Error bars represent standard deviation, *n* ≥ 5. Statistical significance was determined by one-way ANOVA followed by Dunnett’s multiple-comparison test against the positive control PA14. ns = not significant, *p* < 0.0001 = ****.

Having established above that the Δ*pqsH* system was the correct one for our analysis, we proceeded with a second set of exposure experiments. Only PQS was capable of inducing OMV biogenesis under these conditions, and the response was fully restored to wild type levels (Figure 3). The production of OMVs in response to each of the remaining analogs was indistinguishable from that of the uninduced Δ*pqsH* mutant (Figure 3). As in the previous experiment, we again confirmed that SDH activity was undetectable in all OMV samples (Figure S2b). Thus, the loss of any of the PQS functional groups renders the molecule incapable of inducing OMV biogenesis.

### 3.3. MD Simulations Highlight the Differences in Molecular Interactions

To complement our experimental efforts, we performed all-atom molecular dynamics simulations to quantify the interactions between PQS analogs and *P. aeruginosa* outer membranes. Specifically, we characterized the free energy landscape of the molecule–membrane interactions by computing their PMF profiles. The PMF is typically computed as a function of a specific reaction coordinate or collective variable, providing insights into the energetic profile of the system along that particular coordinate. It quantifies relative variations in the free energy of the simulation system as molecules vary their spatial arrangements following the reaction coordinate or collective variable. A reduction in the PMF value indicates a thermodynamically favorable change in the system. Herein, the reaction coordinate represents the molecule’s position in the membrane. We probed two asymmetric OMs with different initial stress states, including zero-leaflet tension (0LT) [87] and surface area matching (SA) [88]. The 0LT membrane was modeled such that the mechanical tension in each leaflet was zero, resulting in zero differential stress (defined as the difference between the two individual leaflet tensions) [49,56]. On the other hand, the SA membrane was constructed to match the surface areas of the two leaflets obtained in the corresponding symmetric bilayers. However, this widely employed building protocol resulted in an overcrowded outer leaflet and a non-zero leaflet tension in this system, as demonstrated in our previous study [89]. The detailed lipid compositions of the two membranes are given in Table S1.

**Figure 4:**
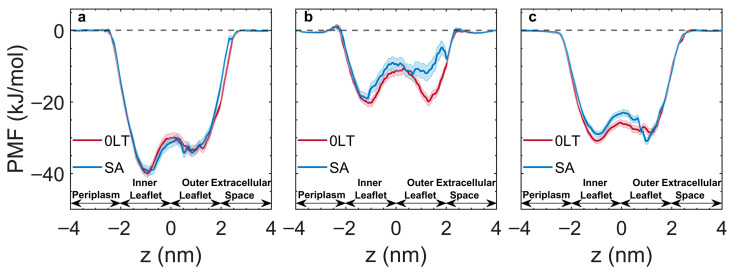
Free energy profiles of alkylated analogs. Transmembrane potential of mean force profiles along the membrane normal direction for (**a**) C7-2N, (**b**) HHQ, and (**c**) PQS interacting with the SA and 0LT membranes. The membrane center of mass is located at z = 0. Shaded error regions represent standard deviations obtained from the bootstrapping method.

The reference point of PMF (0 kJ/mol) corresponded to the molecule in the water region far from the membrane. Figure 4 shows that the major features of the profiles are similar regardless of the interacting molecule and the membrane stress state. Namely, the PMF decreases as the molecule starts interacting with the OM. The profile exhibits a significant decrease within the membrane region with two local minima, and recovers to the zero level when the molecule exits on the inner leaflet side. Despite sharing similar characteristics, these profiles demonstrate interesting differences in the potential well depth and activation energies corresponding to the flip of the small molecule from the outer to the inner leaflet. This energy barrier is calculated as the difference between the energies corresponding to the outer leaflet minimum and the peak at the bilayer midplane.

The low clogP (calculated log octanol:water partition coefficient) values for 2-naphthol, 4-quinolinol, and DHQ (Figure 1) suggest that this set of less hydrophobic analogs would have weak interaction with the hydrophobic membrane due to the lack of an alkyl chain. The PMF profiles exhibited shallow wells in accordance with the clogP values (Figure S3). The experiments above also confirmed that these analogs failed to stimulate OMV formation. Therefore, further computational analysis focused only on the alkylated molecules: C7-2N, HHQ and PQS. C7-2N displays the deepest energy wells compared to PQS and HHQ, consistent with it having the highest clogP (Figure 4a). The profile also suggests a preference for the inner leaflet compared to the outer leaflet. Interestingly, the profiles of C7-2N in the two membranes (0LT vs. SA) were identical regardless of the membrane stress state. In contrast, HHQ exhibited a significant sensitivity to membrane stress (Figure 4b). While its affinity for the two leaflets was the same in the 0LT membrane, HHQ predictively preferred to interact with the undercrowded inner leaflet of the SA membrane. Additionally, the energy barrier for the outer-to-inner flip significantly decreased from $E_{a}^{0LT}=9.2\pm 1.3\;kJ/mol$ to $E_{a}^{SA}=3.1\pm 1.6\;kJ/mol$, indicating a higher probability of HHQ translocation within the SA membrane. The behavior of PQS is different from either C7-2N or HHQ (Figure 4c). The position corresponding to the global energy minimum shifts from the inner leaflet to the outer leaflet upon changing the 0LT membrane to the SA–membrane. The barrier to flip–flop also greatly increases with this change ($E_{a}^{0LT}=2.8\pm 1.3\;kJ/mol$ vs. $E_{a}^{SA}=8\pm 1.3\;kJ/mol).$

### 3.4. Hydrogen Bond Analysis Explains PQS Preference for the Outer Leaflet

We further characterized the molecular interactions between the small molecules and the membrane, focusing on the alkylated compounds C7-2N, HHQ, and PQS. Our analysis determined the number of hydrogen bonds the small molecules formed with membrane lipids as a function of their z-position. Herein, the reported hydrogen bond numbers are averaged over the entire PMF sampling period. The transmembrane hydrogen bond profiles in Figure S4 display two peaks in the outer and inner leaflet regions. The peak positions generally coincide with the local minima in the PMF profile, indicating the preferred interaction sites for these molecules.

**Figure 5:**
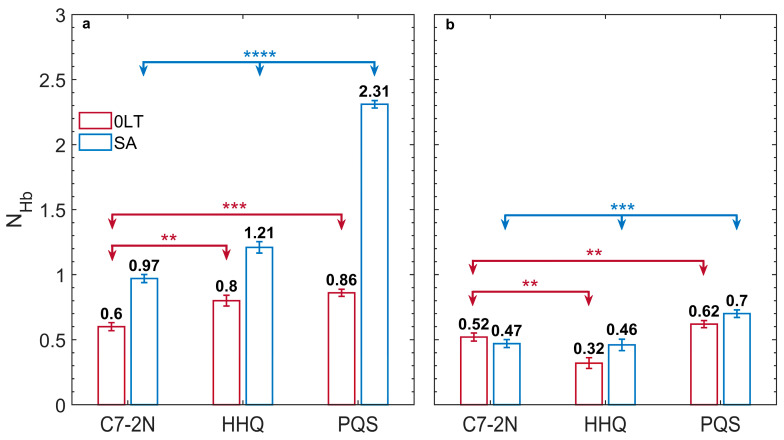
Hydrogen bonding interactions with the inner and outer leaflets. The maximum average number of hydrogen bonds for the molecules in (**a**) the outer leaflet and (**b**) the inner leaflet of the membrane. The donor–acceptor distance of 0.35 nm and an angle of 30° were used as criteria for the formation of hydrogen bonds. Error bars represent the standard error of the mean over time. Statistical significance was determined by one-way ANOVA followed by Tukey’s multiple comparisons test. *p* < 0.01 = **, *p* < 0.001 = ***, *p* < 0.0001 = ****.

To better distinguish and compare the behavior of each molecule, we focused on the maximal number of hydrogen bonds within each leaflet and present it in Figure 5. Compared to C7-2N and HHQ, PQS formed the largest number of hydrogen bonds with the membrane lipids, particularly in the outer leaflet. Moreover, when the outer leaflet became more crowded, as in the SA condition, the number of PQS hydrogen bonds increased from 0.86±0.02 to 2.31±0.02. In contrast, C7-2N and HHQ showed less significant increases in hydrogen bonds with Lipid A in the SA membrane compared to the 0LT membrane. Notably, C7-2N and HHQ have exactly complementary functional groups to recover those of PQS. However, the increase in the number of hydrogen bonds for PQS (1.45±0.03) significantly exceeded the summation of the increases for C7-2N and HHQ (0.78±0.07). For all three molecules, Figure 5 shows considerably fewer hydrogen bonds formed with phospholipids in the inner leaflet.

## 4. Discussion

Across species, OMVs continue to be implicated in toxin trafficking, immune evasion, cell–cell communication, and more [5]. It is therefore critical we understand the mechanisms that bacteria use to produce and deliver these versatile structures. Our group developed the Bilayer Couple model to describe OMV production in response to membrane perturbation by small molecules like PQS [48]. Recently, we also identified the potential involvement of the ring nitrogen of PQS in interactions that facilitate membrane insertion and the initiation of OMV biogenesis [63]. With this work, we explore the hypothesis that the PQS ring nitrogen is essential for OMV stimulation and employ MD simulation to help understand the nature of the interactions PQS functional groups make with membrane lipids to drive this important process.

We first set out to test the ability of PQS analogs missing specific functional groups to stimulate OMV biogenesis in the Δ*pqsA* mutant, which is incapable of synthesizing its own PQS. The mutation in this strain knocks out the first step in the production of a wide variety of 4-hydroxy-2-alkyl-quinoline (HAQ) secondary metabolites produced by *P. aeruginosa* [85] (including PQS). The final step of PQS biogenesis is carried out by the product of the *pqsH* gene [86]. In the first analysis of HAQ ability to stimulate OMV biogenesis, Mashburn et. al. established that HHQ lacked OMV-inducing capabilities by exogenously adding it to the Δ*pqsA* Δ*pqsH* double mutant [61]. Early in the analysis, we observed that exogenously added HHQ can be converted into PQS by the Δ*pqsA* single mutant. This was evidenced both by the partial rescue of OMV production by Δ*pqsA* + HHQ and the direct measurement of PQS production in the culture. This result demonstrates that HHQ can be imported into the cell and acted upon by the PqsH enzyme to produce PQS, which is then capable of inducing OMV biogenesis. The existence of a similar cross-feeding ability was observed by Déziel et al. [85] in the context of PQS quorum sensing. The results here indicate that PQS produced from precursor cross-feeding also retains its ability to stimulate OMV biogenesis. Combined with our previous finding that PQS must be properly exported in order to induce OMV biogenesis [58], this highlight a major gap in knowledge surrounding the mechanisms by which HHQ and PQS are transported across membranes. It has been suggested that MexEF-OprN is involved in the export of HHQ [90], but import of HHQ and transport of PQS in either direction remain to be characterized. These are challenging questions due to the array of small-molecule export systems employed by *P. aeruginosa*, but they must be tackled if we are to fully understand quorum sensing and virulence in this clinically important organism.

Once we established that the Δ*pqsH* background was the most appropriate for our analysis, we continued investigating structure–activity relationships in PQS and its analogs. HHQ and PQS analogs with progressively shorter alkyl chains were previously studied [61], and this highlighted the importance of the hydroxyl group and alkyl chain for PQS-induced OMV formation. The Bilayer Couple model of OMV biogenesis [48] predicts that loss of OMV inducing capability may be the result of reduced interaction with Lipid A in the outer leaflet of the outer membrane. To investigate this, we built an MD simulation of the *P. aeruginosa* OM [91] and used it to interrogate PQS–Lipid A interactions [63]. This analysis confirmed the importance of the hydroxyl and alkyl groups, but also suggested a previously unknown role for the ring nitrogen group in binding LPS phosphates to facilitate PQS insertion into the membrane [63]. With the current work, we aimed to conduct a systematic analysis of PQS analogs lacking one or more important functional group. In both the Δ*pqsA* and Δ*pqsH* backgrounds, we demonstrated that all PQS functional groups were required for a molecule to stimulate OMV formation. This validates the MD simulation prediction of the importance of the PQS ring nitrogen [63], and highlights the utility of in silico modeling as a tool to guide mechanistic experiments in the laboratory. Further, the fact that the analogs failed to stimulate OMV biogenesis in both genetic backgrounds reinforces the fact that no other HAQ produced by *P. aeruginosa* is capable of inducing OMV production and that, aside from HHQ, no analog was able to be converted into PQS by either PqsH or the products of the *pqsABCDE* operon.

Having demonstrated that the ring nitrogen is critical for PQS function, we returned to MD simulation to try to understand why the presence of all PQS functional groups together is so advantageous. Simulations using 2-naphthol, 4-quinolinol, and DHQ showed that these molecules interacted less strongly with the membrane. This is consistent with each of these molecules lacking the alkyl group and having lower clogP than the other analogs. The free energy profiles of PQS, HHQ, and 3-heptyl-2-naphthol (C7-2N) indicate that the insertion of all three molecules into the membrane is energetically favorable. The strong hydrophobic nature of C7-2N contributes to it showing the highest affinity for the membrane. However, as C7-2N prefers the phospholipid inner leaflet regardless of the membrane stress state, it would be incapable of inducing positive curvature and membrane budding. The simulation reveals that the interactions of HHQ and PQS with lipids are more sensitive to membrane stress, likely attributed to the ketone and pyridinic nitrogen serving as hydrogen bond acceptors. The results indicate that mechanical stress within the bilayer not only influences the molecules’ preference for a specific leaflet but also modulates their tendency to flip-flop. It is noteworthy that PQS was the only analog that showed a preference for the outer leaflet when it became crowded. Moreover, the activation energy corresponding to PQS flipping from the outer to the inner leaflet also greatly increased. Together, these changes would likely lead to the accumulation of PQS in the outer leaflet under these conditions.

The molecular basis for PQS preference for the outer leaflet was revealed when we analyzed the hydrogen bonding potential of PQS, HHQ and C7-2N in both the 0LT and SA membranes. With its greater number of hydrogen bonding functional groups, PQS was much more likely to engage in multiple simultaneous hydrogen bonds to Lipid A than the other two analogs. Intriguingly, the functional groups in PQS exhibited a synergy in promoting hydrogen bond formation in response to membrane stress beyond a simple additive effect. Despite the lack of a direct correlation with the PMF profile, this synergistic hydrogen bond formation could contribute to PQS’s unique stress-induced preference for the outer leaflet.

We previously showed that insertion of PQS into the outer leaflet increased lateral pressure and crowded the membrane lipids there [89]. Here, we demonstrate that PQS is the only analog where crowdedness increased the preference of the molecule for the outer leaflet, most likely by enabling maximal hydrogen bonding engagement with Lipid A. Thus, the insertion of PQS into the outer leaflet of the OM encourages more PQS to locally accumulate, increasing differential leaflet stress until membrane curvature is induced. This set of events is in agreement with the Bilayer Couple model [48], and would explain why PQS is found concentrated in released OMVs both in *P. aeruginosa* cultures [16] and when exogenously added as an OMV inducer to other species [59,60].

## 5. Conclusions

In this study, we set out to characterize the interactions between a small-molecule inducer of OMV biogenesis and the lipids within the membrane. We wanted to understand whether specific interactions between PQS and Lipid A could explain the ability of this molecule to induce membrane curvature while similar compounds do not. Building upon previous structure–activity analyses, we discovered that the ring nitrogen of PQS is required for OMV-stimulation. In fact, we demonstrated that the hydroxyl group, alkyl chain and ring nitrogen functional groups must all be present for PQS function. The reason for this became clear when we simulated the interactions of PQS and its analogs with membrane lipids. PQS engaged in significantly more interactions with Lipid A than any of its analogs, contributing to a preference for interaction with the outer leaflet of the outer membrane. Interestingly, this advantage was enhanced as the leaflet became more crowded. Thus, the unique structure of PQS allows it to preferentially interact with the outer leaflet of the membrane, initiating the crowding that will facilitate the accumulation of more PQS. This positive feedback mechanism is wholly consistent with the Bilayer Couple model and explains on a molecular level how PQS drives the formation of OMVs. Gaining a deeper understanding of the mechanisms bacteria use to deliver toxins and thwart the immune system is a critical step toward the development of new treatments.

## Data Availability

The original contributions presented in this study are included in the article/Supplementary Materials. Further inquiries can be directed to the corresponding author(s).

## Supplementary Materials

The following supporting information can be downloaded at: https://www.mdpi.com/article/10.3390/membranes15080247/s1, Figure S1: Exogenous addition of PQS analogs to Δ*pqsA*; Figure S2: Analysis of SDH activity in OMV samples; Figure S3: Transmembrane potential of mean force profiles along the membrane normal direction for non-alylated analogs of PQS interacting with the 0-LT membrane; Figure S4: Time-averaged number of hydrogen bonds for (a) C7-2N, (b) HHQ and (c) PQS; Table S1: Molecular composition of the outer membranes in MD simulations.
