# Supplementary material for: Membrane Stress Enhances Specific PQS–Lipid Interactions That Drive Bacterial Outer Membrane Vesicle Biogenesis
Source: Membranes (Basel). 2025 Aug 13;15(8):247. doi: 10.3390/membranes15080247 (PMC12388362; doi:10.3390/membranes15080247)
Supplement: Supplementary file 1 [file membranes-15-00247-s001.zip › membranes-3685394-supplementary.pdf]

Supplemental Material for:

Membrane stress enhances specific PQS-lipid interactions that drive outer membrane vesicle biogenesis.

Citrupa Gopal, Hasan Al Tarify, Emad Pirhadi, Eliza G. O'Brien, Anuradha Dagar, Xin Yong, Jeffrey W. Schertzer*

SUPPLEMENTAL METHODS:

Synthesis of 3-heptyl-2-naphthol.

Unless specified, all reagents and starting materials were purchased from commercial sources and used as received without purification. Flash chromatography was performed using silica gel (230–400 mesh) with hexanes, ethyl acetate, and diethyl ether as eluents. All reactions were monitored by thin-layer chromatography (TLC) and gas chromatography (GCMS-QP2020). ^{1}H ^{13}C NMR spectra were recorded on a 400 MHz Bruker NMR spectrometer and were described as chemical shifts in ppm, multiplicity (s, singlet; d, doublet; t, triplet; q, quartet; doublet–doublet (dd), pentet (p), m, multiplet), coupling constant in hertz (Hz), and number of protons.

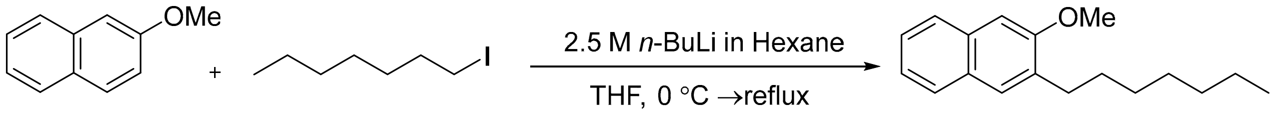

1 2 3

General procedure for the synthesis of 3-*n*-heptyl-2-methoxynaphthalene (3). *n*-Butyl lithium (2.5 M in hexane (2.2mmol, 1.1 equiv.) was added to a stirring solution of 2-methoxynaphthalene (316 mg, 2.0 mmol, 1.0 equiv.) in dry THF (10 ml) at 0°C under argon. The mixture was stirred at 0°C for 30 minutes, followed by reflux for 1 hr. A solution of *n*-heptyl iodide (360µl, 2.2 mmol, 1.1 equiv.) in dry THF (2 ml) was then added dropwise and refluxing was continued for another 2 hr. The cooled mixture was poured onto an ice water bath (20 ml) and saturated $NaHCO_3$ (2 x 100 ml), then extracted with ethyl acetate (3 x 5 ml). The reaction mixture was then concentrated under reduced pressure and purified by silica gel column chromatography (EtOAc/hexane = 1:99) to afford compound **3**.
The residue was further purified by column chromatography using 2% ethyl acetate and hexane as the eluent to afford 471 mg (91%) of colorless oil of compound **3**.

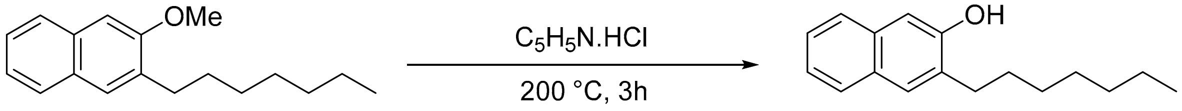

3 4

General procedure for the synthesis of 3-*n*-Heptyl-2-naphthol (4). A mixture of **3** (256 mg, 1.0 mmol, 1.0 equiv.) and dry pyridine·HCl (138.0 mg, 1.2 mmol) was heated with stirring at 200°C for 2 hr. After being cooled to about 100°C, the mixture was poured into ice water (2.0 ml) and extracted with benzene (2 x 5 ml). The combined organic layers were washed with water, dried, and evaporated. Distillation of the residue gives a colorless oil which solidified on standing in the cold. Recrystallization from *n*-pentane afforded 153 mg (60%) of colorless compound **4**.

3-*n*-heptyl-2-methoxynaphthalene colorless oil;

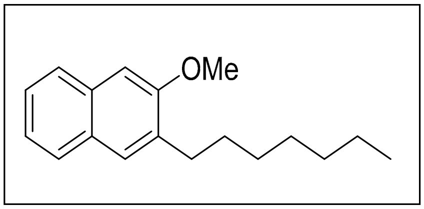

^{1}H NMR (400 MHz, CDCl$_3$) δ$_H$ 7.80 (d, *J* = 8.6 Hz, 2H), 7.65 (s, 1H), 7.47 (t, *J* = 7.5 Hz, 1H), 7.40 (t, *J* = 6.9 Hz, 1H), 7.16 (s, 1H), 4.00 (s, 3H), 2.84 – 2.88 (m, 2H), 1.74 – 1.81 (m, 2H), 1.37 – 1.52 (m, 8H), 1.00 (t, *J* = 6.9 Hz, 3H);
^{13}C NMR (100 MHz, CDCl$_3$) δ$_C$ 156.6, 133.3, 133.1, 128.9, 127.9, 127.0, 126.3, 125.4, 123.4, 104.7, 55.2, 31.9, 30.7, 29.8, 29.6, 29.3, 22.7, 14.2.

3-*n*-Heptyl-2-naphthol brown solid;

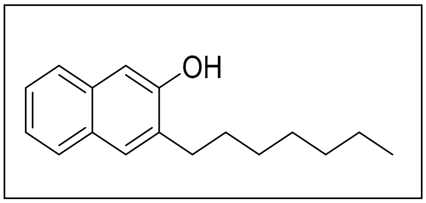

^{1}H NMR (400 MHz, CDCl$_3$) δ$_H$ 7.71 (d, *J* = 8.2 Hz, 1H), 7.63 (d, *J* = 8.0 Hz, 1H), 7.58 (s, 1H), 7.36 (t, *J* = 7.5 Hz, 1H), 7.30 (t, *J* = 7.5 Hz, 1H), 7.09 (s, 1H), 4.89 (s, 1H), 2.75 – 2.79 (m, 2H), 1.67 – 1.74 (m, 2H), 1.28 – 1.44 (m, 8H), 0.89 (t, *J* = 6.9 Hz, 3H);
^{13}C NMR (100 MHz, CDCl$_3$) δ$_C$ 152.5, 133.2, 132.3, 131.1, 129.2, 128.6, 127.2, 125.8, 123.5, 109.3, 34.2, 30.6, 29.7, 29.5, 29.3, 22.4, 14.1.

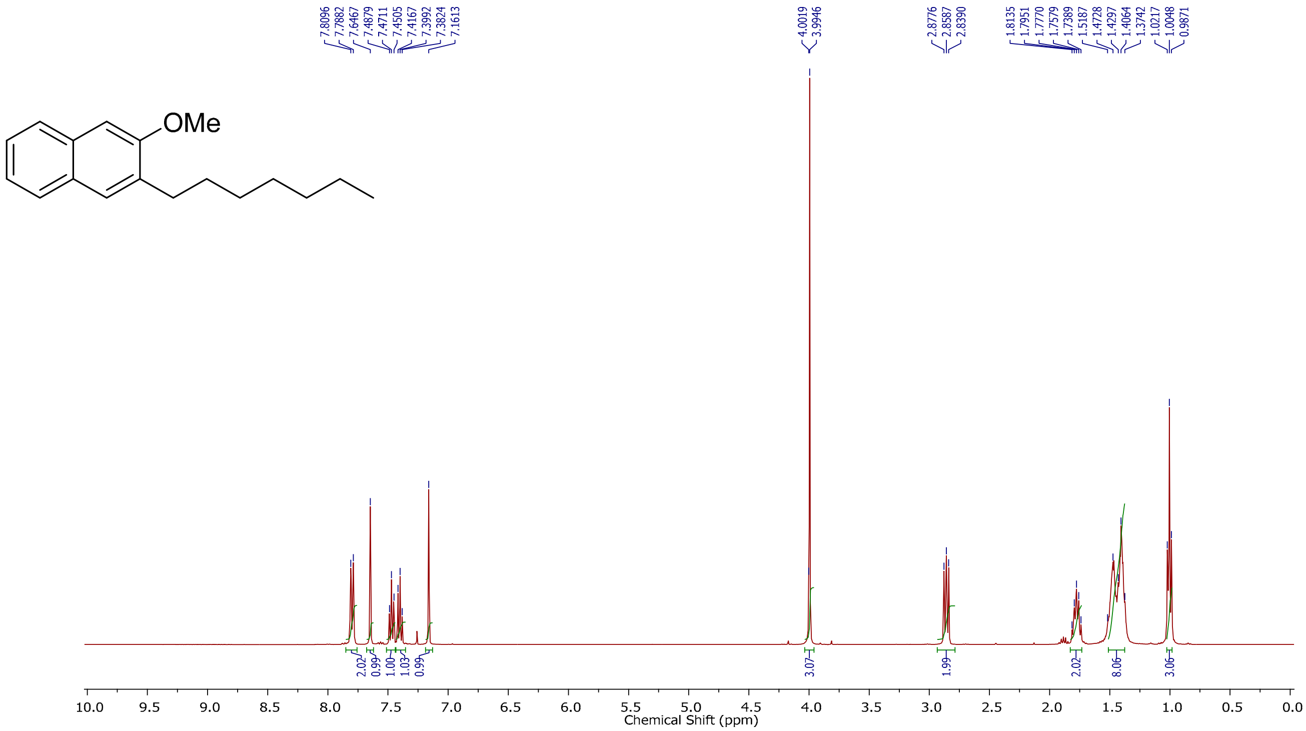

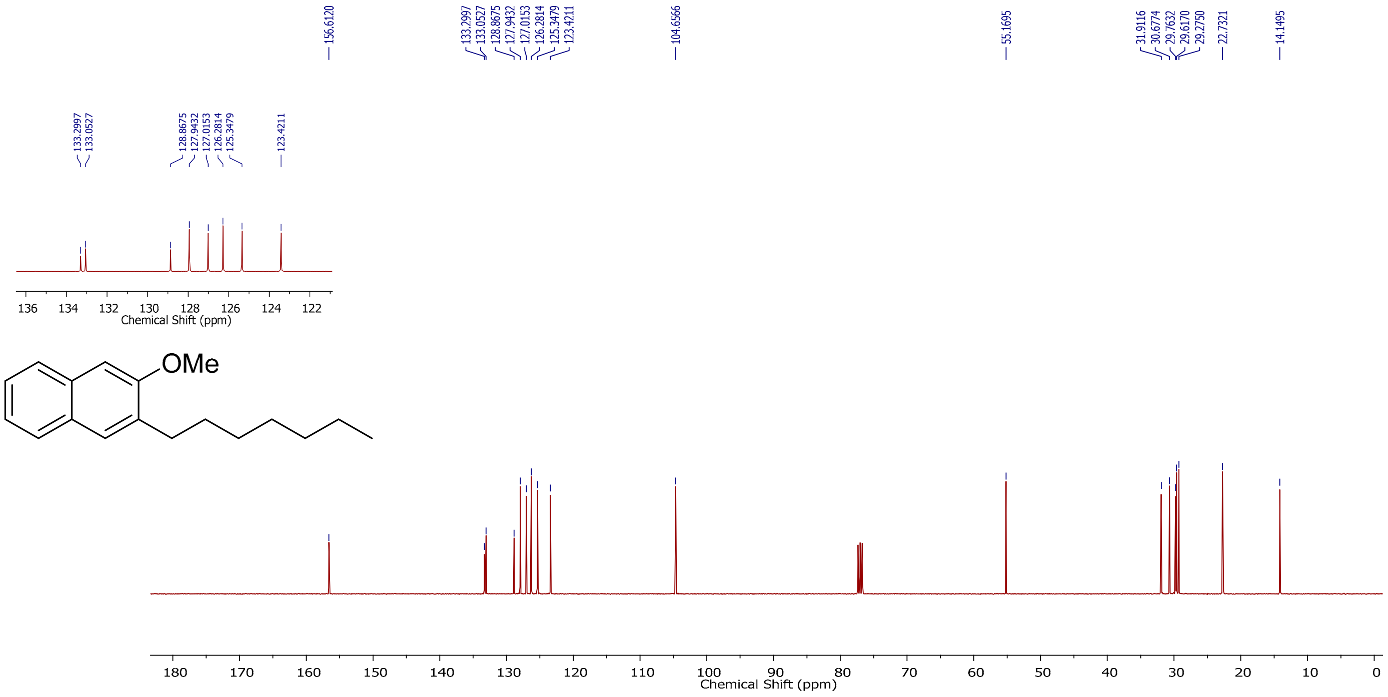
156.6120
133.2997
133.0527
128.8675
127.9432
127.0153
126.2814
125.3479
123.4211
104.6566
55.1695
31.9116
30.6774
29.7632
29.6170
29.2750
22.7321
14.1495
133.2997
133.0527
128.8675
127.9432
127.0153
126.2814
125.3479
123.4211
136 134 132 130 128 126 124 122
Chemical Shift (ppm)
OMe
180 170 160 150 140 130 120 110 100 90 80 70 60 50 40 30 20 10 0
Chemical Shift (ppm)

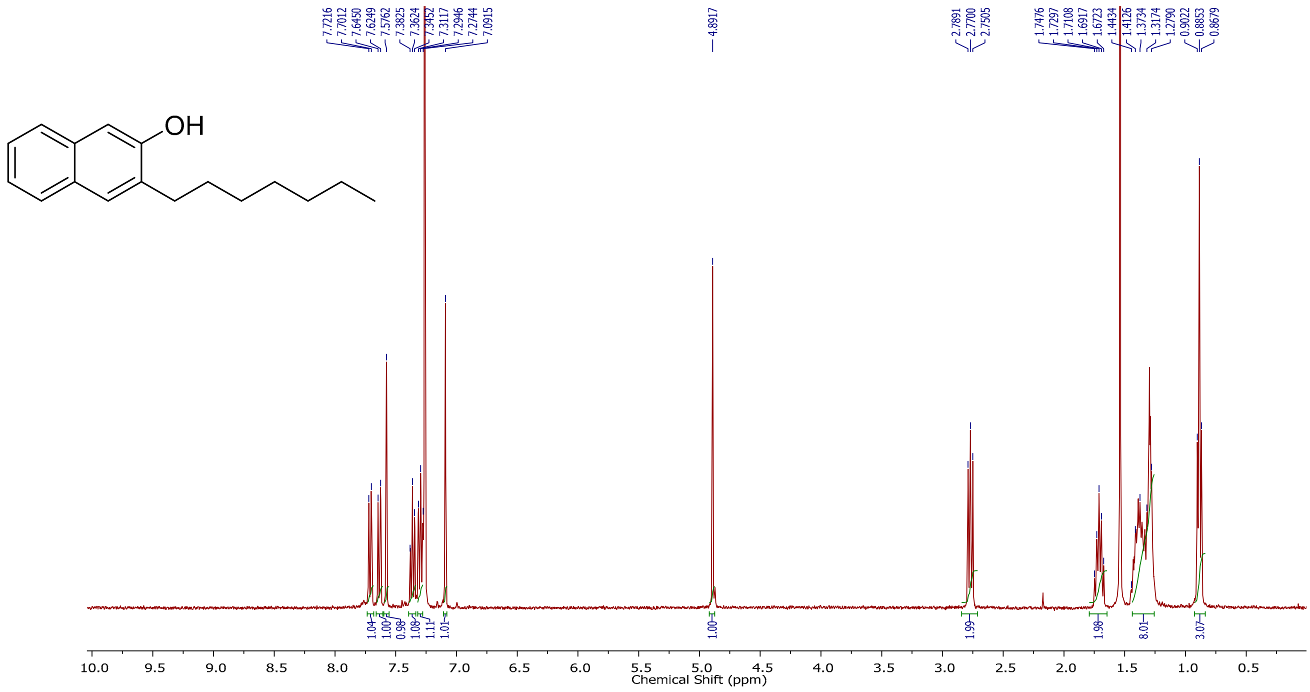
7.7216
7.7012
7.6450
7.6249
7.5762
7.3825
7.3624
7.3452
7.3117
7.2946
7.2744
7.0915
4.8917
2.7891
2.7700
2.7505
1.7476
1.7297
1.7108
1.6917
1.6723
1.4434
1.4126
1.3734
1.3174
1.2790
0.9022
0.8853
0.8679
OH
1.04
1.00
0.99
1.08
1.11
1.01
1.00
1.99
1.98
8.01
3.07
10.0 9.5 9.0 8.5 8.0 7.5 7.0 6.5 6.0 5.5 5.0 4.5 4.0 3.5 3.0 2.5 2.0 1.5 1.0 0.5
Chemical Shift (ppm)

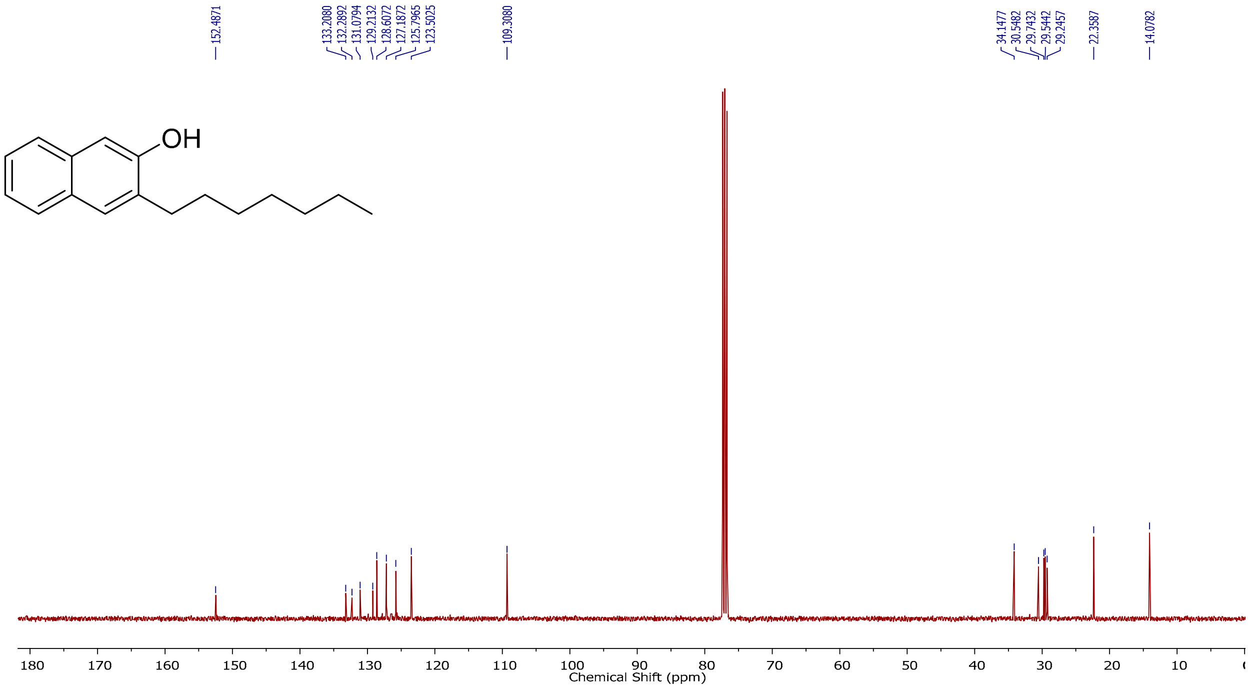
OH
152.4871
133.2080
132.2892
131.0794
129.2132
128.6072
127.1872
125.7965
123.5025
109.3080
34.1477
30.5482
29.7432
29.5442
29.2457
22.3587
14.0782
180
170
160
150
140
130
120
110
100
90
80
70
60
50
40
30
20
10
Chemical Shift (ppm)

SUPPLEMENTAL TABLES:

Table S1. Molecular composition of the outer membranes in MD simulations.

**Membrane type**	Lipid per leaflet		Water
	Outer (LPA)	Inner (POPE-POPG)	
SA	35	64-29	8716
0LT	39	73-33	9197

SUPPLEMENTAL FIGURES:

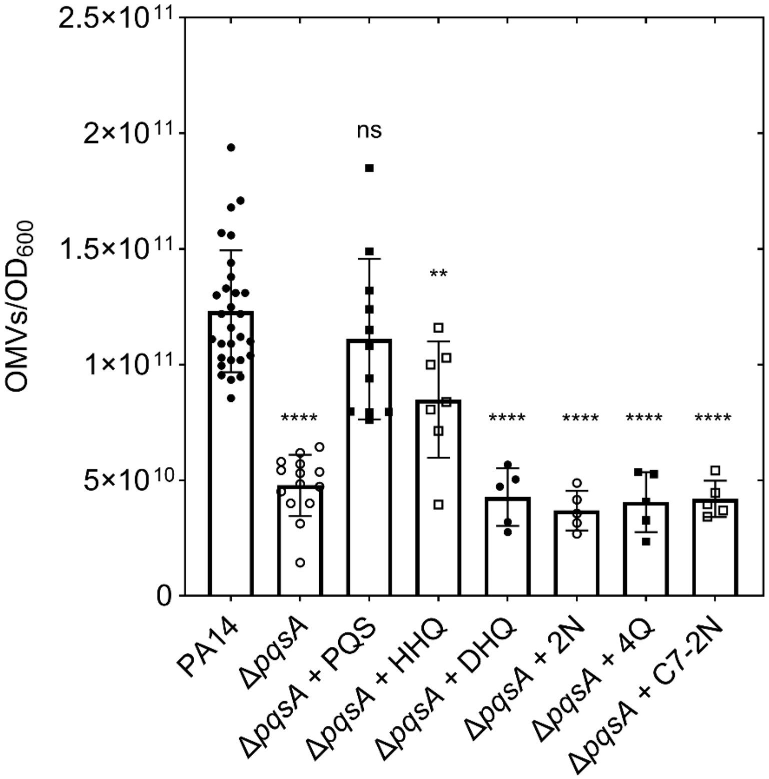

Fig S1. Exogenous addition of PQS analogs to Δ*pqsA*. All samples were incubated for 4 hours at 37°C post treatment, followed by OMV harvesting and quantification by Nanoparticle Tracking Analysis. Error bars represent standard deviation. Statistical significance was determined by One-way ANOVA followed by Dunnett's multiple comparisons test against the positive control PA14. ns = not significant, $p<0.05$ = *, $p<0.01$ = **, $p<0.001$ = ***, $p<0.0001$ = ****. $n \geq 5$.

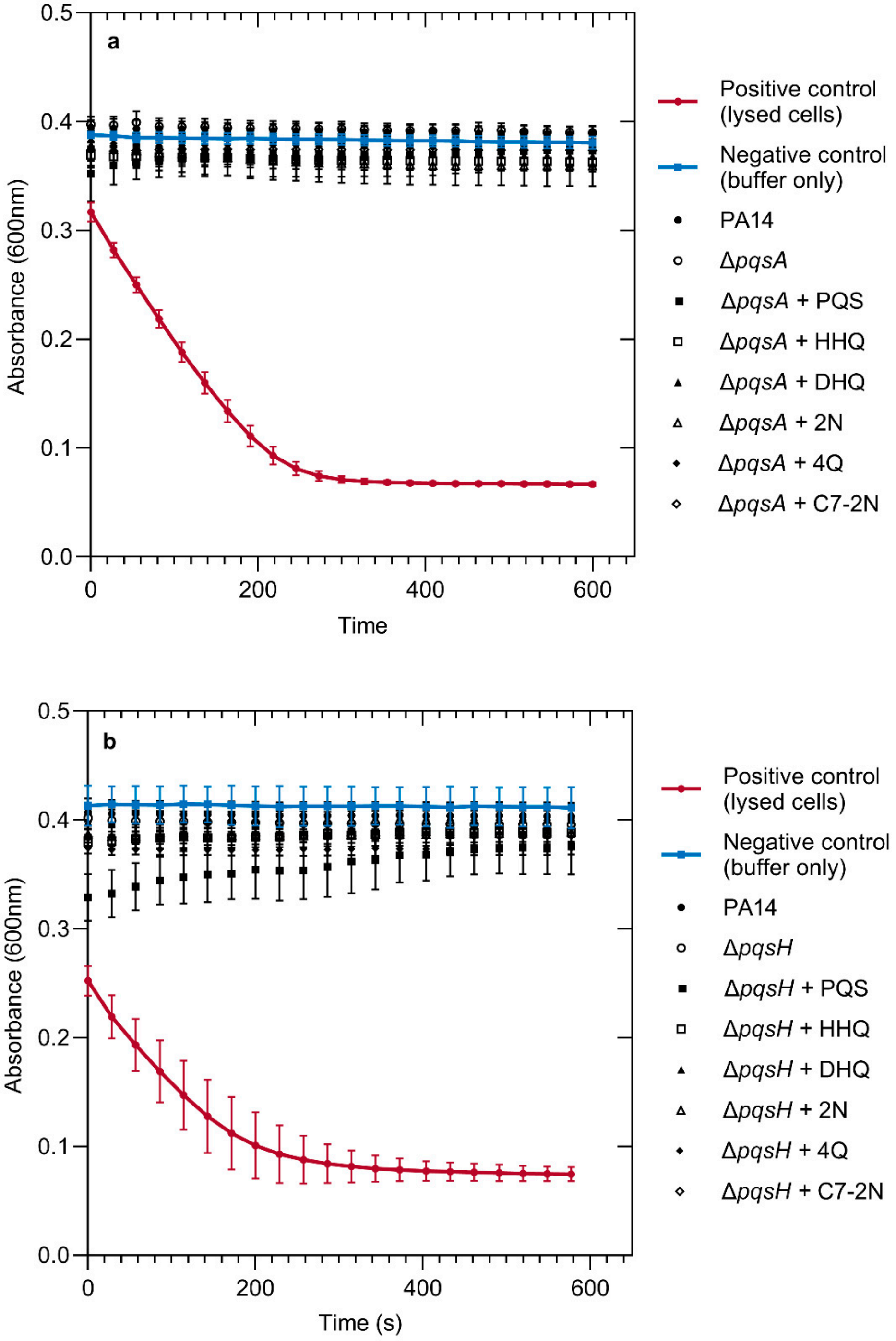

Fig S2. Analysis of SDH activity in OMV samples. OMV samples were routinely tested for SDH activity as described in Materials and Methods. All samples shown in Figures 2, 3 and S1 were assessed in this way. The above is a representative example of one biological replicate done in triplicate for a) Δ*pqsA* and b) Δ*pqsH*.

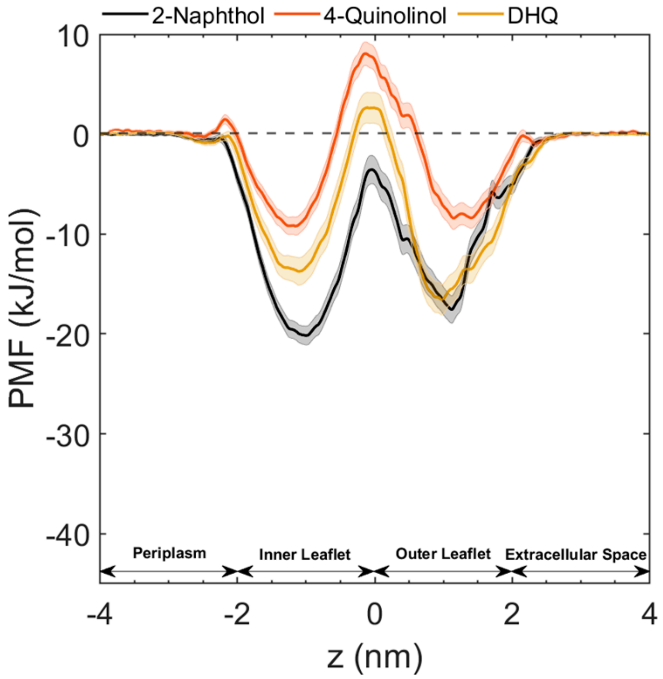

Figure S3: Transmembrane potential of mean force profiles along the membrane normal direction for non-alylated analogs of PQS interacting with the 0-LT membrane. The membrane center of mass is located at z = 0. Shaded error regions represent standard deviations obtained from bootstrapping method.

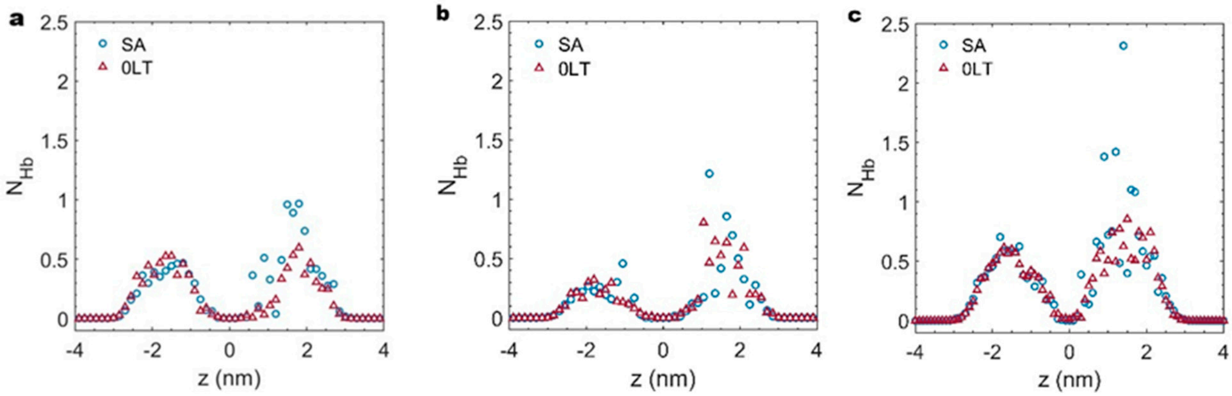

Figure S4: Time-averaged number of hydrogen bonds for a) C7-2N, b) HHQ and c) PQS. Hydrogen bonds are formed at a distance of 0.35 nm and an angle of 30°.
